# Putative sex pheromone of the Asian citrus psyllid, *Diaphorina citri*, breaks down into an attractant

**DOI:** 10.1038/s41598-017-18986-4

**Published:** 2018-01-11

**Authors:** Odimar Z. Zanardi, Haroldo X. L. Volpe, Arodi P. Favaris, Weliton D. Silva, Rejane A. G. Luvizotto, Rodrigo F. Magnani, Victoria Esperança, Jennifer Y. Delfino, Renato de Freitas, Marcelo P. Miranda, José Roberto P. Parra, José Mauricio S. Bento, Walter S. Leal

**Affiliations:** 1 Research and Development Department, Fund for Citrus Protection (Fundecitrus), Vila Melhado, 14807-040 Araraquara, São Paulo Brazil; 2 Department of Entomology and Acarology, Luiz de Queiroz College of Agriculture, University of São Paulo (USP), Piracicaba, SP 13418-900 Brazil; 3 Department of Molecular and Cellular Biology, University of California-Davis, Davis, CA 95616 USA

## Abstract

Under laboratory conditions, mating activity in Asian citrus psyllid (ACP) started 4 days after emergence, peaked at day 7, and showed a clear window of activity starting 8 h into the photophase and extending through the first hour of the scotophase. We confirmed that ACP males are attracted to emanations from conspecific females. Traps loaded with a candidate compound enriched with female extract, lignoceryl acetate (24Ac), at various doses were active only after being deployed for several weeks in the field, suggesting that a degradation product, not the test compound, was the active ingredient(s). Lignocerol, a possible product of 24Ac degradation, was not active, whereas acetic acid, another possible degradation product, was found in the airborne volatile collections from lures matured under field conditions and detected in higher amounts in volatiles collected from females at the peak of mating activity than in male samples. Acetic acid elicited dose-dependent electroantennographic responses and attracted ACP males, but not females, in Y-type and 4-way olfactometers. Field tests showed that acetic acid-baited traps captured significantly more males than control traps. Surprisingly, captures of females in acetic acid-baited traps were also higher than in control traps, possibly because of physical stimuli emitted by captured males.

## Introduction

The Huanglongbing (HLB), also known as citrus greening, is one of the most devastating problems in agriculture worldwide, particularly for the citrus industry^1^ given that, once infected, trees must be eradicated. In Brazil, as many as 46.2 million citrus trees (representing 26% of the currently planted trees) have been eradicated since the detection of HLB in 2004^2^. In Florida, HLB has caused severe losses to the citrus industry. Because of HLB and hurricane Irma, this year’s production is forecast to be 68.7 million boxes of oranges, as compared to 96.9 million boxes produced in 2014-2015^3^. HLB is caused by endogenous, phloem-restricted bacteria of the genus *Candidatus* Liberibacter spp., which are transmitted from tree to tree by the Asian citrus psyllid, *Diaphorina citri* Kuwayama (Hemiptera: Liviidae) in Asia and America and the African citrus psyllid, *Trioza erytreae* (Del Guercio) (Hemiptera: Triozidae) in Africa^1^. Two other psyllid species have been implicated without actual transmission tests^4^. Thus, the Asian citrus psyllid (ACP), which led to HLB being widespread in China, Brazil, and the United States^4^, is today’s most serious threat to the citrus industry. In places like Arizona and California where ACP is present, but the disease apparently has not been established, the emphasis is on early detection, eradication, and limiting the spread of the disease^4^, whereas in other areas like in Florida, where HLB is widespread^4^, monitoring ACP populations is essential to avoid reinfection after eradication of infected plants. Currently, colored sticky traps are widely used for *D*. *citri* detection and population monitoring in field studies^5^. Efficient lures are sorely needed for sticky traps, particularly for early ACP detection; otherwise, farmers have to resort to regular sprays to avoid infection given that infected insects from gardens and noncommercial areas migrate to citrus farms^6^. Pheromones and other semiochemicals have been widely used in agriculture and medical entomology as lures in trapping systems for monitoring and surveillance^7^, as well as strategies for controlling populations, such as mating disruption^8^ and attraction-and-kill systems^9^.

Although the superfamily Psylloidea includes more than 3800 described species, males of only 4 species have been shown to be attracted to “pheromones” produced by conspecific females^10^, specifically males from *Cacopsylla bidens*, *C*. *pyricola* ^11^, *Bactericera cockerelli* ^12^, and *D*. *citri* ^13^. Subsequently, Mann *et al*. identified cuticular hydrocarbons from *D*. *citri* females, which were attractive to conspecific males in laboratory assays, but did not increase total trap catches as compared to blank traps under field conditions^14^. Hitherto, ACP pheromones remain elusive, most probably because ACP’s complex behavior and biology have confounded isolation of the active semiochemicals. These confounding factors include acoustic communication^15^, the unusual observation that mated females appear more attractive than virgin females^13^, influence of the female abdomen color on male attraction^16^, and the intriguing report that male attraction to female odor significantly increased after mating experience^17^. We aimed at the identification of ACP-derived semiochemicals for possible applications as trap lures, but given the scenario in the current literature and due to possible geographical variations, we decided to start from scratch by establishing a laboratory colony of ACP with insects collected on orange jasmine (Brazilian common name, murta), *Murraya paniculata*, in São Paulo, Brazil, studying its basic biology and behavior, attempting to isolate and identify sex-specific or sex-enriched semiochemicals, and testing them under laboratory and field conditions. Here, we report on the identification of an ACP sex pheromone, which was initially detected while testing another putative sex pheromone under field conditions.

## Results and Discussion

### Mating behavior

**Figure 1:**
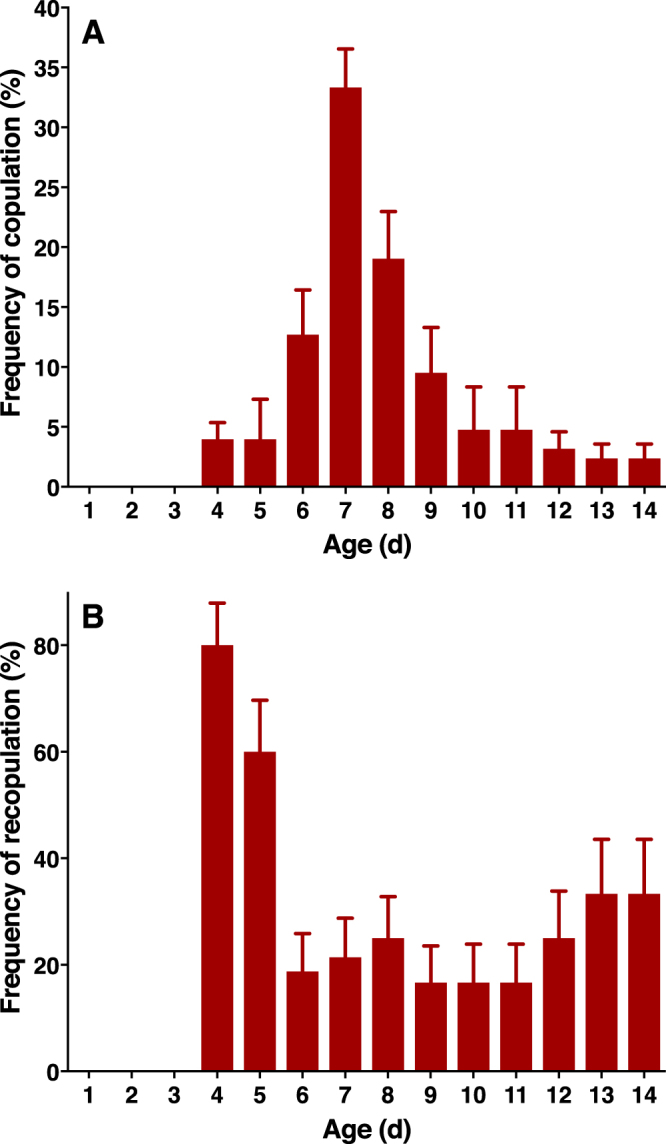
Mating behavior of the Asian citrus psyllid. (**A**) Age dependence on the frequency of copulation. (**B**) Frequency of recopulation, according to insect age.

**Figure 2:**
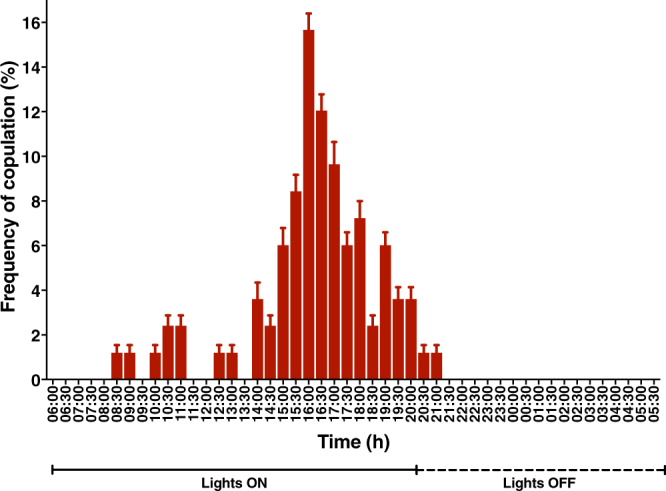
Diel rhythm of mating activity. Seven-day-old adults showed a window of mating activity towards the end of the scotophase, with a peak corresponding to the peak of flight activity under field conditions.

Using our laboratory colony, we studied ACP mating behavior when individual couples were kept in the laboratory on a common host, *M*. *paniculata*, and under conditions of photoperiod, luminosity, relative humidity, and temperature mimicking those found under natural conditions in most citrus fields in Brazil. As opposed to what has been observed^18^ with a laboratory colony originated from ACP collected in Florida in early 2000^19^, no mating activity occurred in the first 3 days after emergence (Fig. 1A). Mating activity started 4 days after emergence, reached a clear peak with 7-day-old insects, and declined thereafter (Fig. 1A). Interestingly, couples that copulated earlier than the peak time of copulation (7 days) showed the highest frequency of re-copulation, particularly those mating 4- or 5-days after emergence (Fig. 1B). These findings suggest that earlier mating is not as efficient as mating at or after the peak of mating activity. Apparently, the frequency of remating was not influenced by the duration of copulation, given that mating by 4- to 14-day-old couples lasted nearly the same time and were not significantly different (4d, 43.3 ± 5.27 min, mean ± SEM; 5d, 41.3 ± 5.49 min; 6d, 45.8 ± 3.53 min; 7d, 55.9 ± 3.15 min; 8d, 58.0 ± 3.19 min; 9d, 60.0 ± 6.29 min; 10d, 51.4 ± 5.53 min; 11d, 38.6 ± 5.53 min; 12d, 42.0 ± 7.35 min; 13d, 37.5 ± 10 min; 14d, 37.5 ± 10 min; Tukey’s multiple comparisons test, P = 0.4098–0.9999) (Fig. S1). Next, we studied the diel rhythm of mating activity by 7-day-old couples. It has been reported that in a laboratory colony from Florida, mating occurs throughout daylight hours without an obvious peak of mating activity^18^. By contrast, we observed a clear window of activity towards the end of the photoperiod until the beginning of the photoperiod, starting at 14:00 (ie, 8 h into the photophase) and ending at 21:00 (ie, 1 h after the scotophase started) (Fig. 2). Of note, the reported peak of flight activity under field conditions^20,21^, is contained within the window of mating activity observed under laboratory conditions (Fig. 2). We, therefore, used 7-day-old insects in subsequent studies and measured behavior and performed pheromone extractions during the last 6 h of the photophase.

### Female-produced Sex Pheromone and Male Response

**Figure 3:**
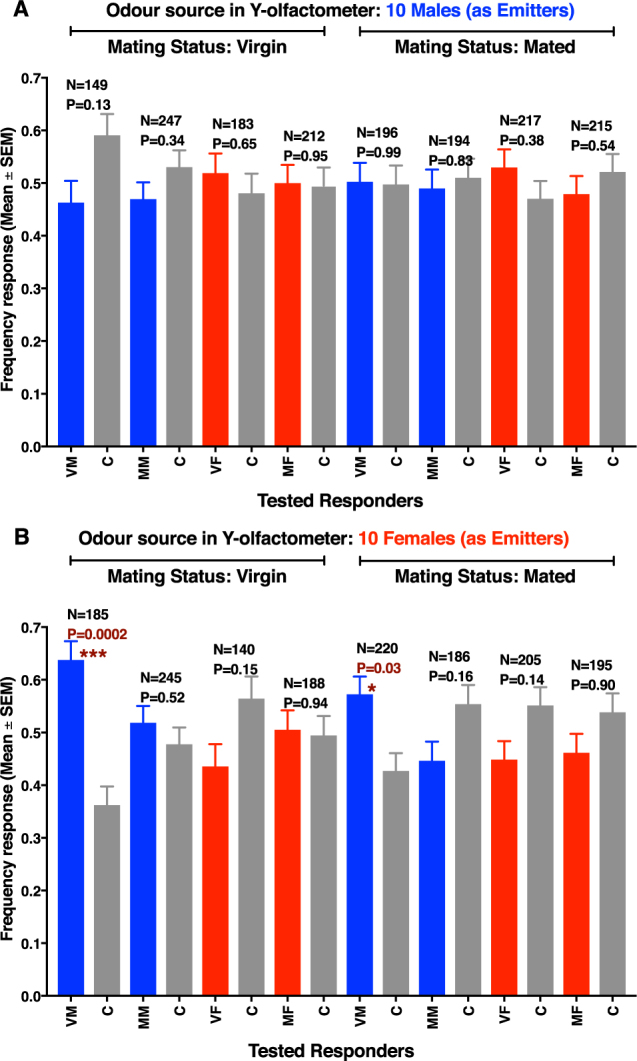
Behavioral responses from ACP males and females to odorants from virgin or mated males and females. (**A**) Males (unmated, left; or mated, right) were used as odor sources. (**B**) Females (virgin, left; or mated, right) were used as odor sources. In both cases, tested responders were virgin males (VM), mated males (MM), virgin females (VF), and mated females (MF). Control (**C**). Mated males and females were used at least 1 day post mating.

Behavioral evidence has been previously provided to demonstrate that *D*. *citri* females produce a sex-pheromone, but intriguingly it has been suggested that mated females appeared to be more attractive than virgin females^13^. Using an olfactometer, we measured the behavioral responses of ACP males or females as responders to odor sources emitted by virgin or mated males, and virgin or mated females. We did not observe any evidence for male or female attraction when either virgin or mated males were used as odor sources (emitters) (Fig. 3A). There were no significant differences between the various treatments and their respective controls when the odor sources were virgin or mated males and the tested responders were virgin males (VM), virgin females (VF), mated males (MM), or mated females (MF). By contrast, when either virgin or mated females were used as odor sources (emitters), virgin males (VM) showed a significant preference for the treatment side of the arena, regardless of the source being virgin or mated females (Fig. 3B). However, when mated males were tested as possible responders, they did not show a significant preference for treatment vs. control when virgin or mated females were used as odor sources. Our findings differ from those observed with a laboratory colony from Florida indicating that male attraction to female-released odorants increased after mating experience^17^, possibly due to the difference in experimental protocols, insect age, time of the tests, and/or possible geographical variations. In sum, under our experimental protocol considering the optimal age of test insects and timing of the bioassays (to coincide with the peak of mating activity), only VM responded to female-released odorants regardless of the emitters’ mating status.

### Attempts to Identify Constituent(s) of the Female-released Sex Pheromone

Having confirmed that odorants released from virgin or mated females attracted virgin males (Fig. 3B), we proceeded towards the isolation and identification of the sex pheromone constituent(s). Initial attempts to extract the active ingredient(s) using airborne volatile collections were unrewarding. Behavioral measurements with airborne volatiles collected from virgin females during the time corresponding to the peak of mating activity did not show a significant preference for treatment vs. control (extracts from headspace collections, 0.51 ± 0.07; control 0.49 ± 0.07; *n* = 53; P = 0.91), possibly because the active ingredients were not trapped under our experimental conditions. We then changed our approach to attempt to isolate the active ingredient(s) by direct solvent extraction. When tested in our Y-olfactometer, males responded significantly to the side arm with a hexane extract from virgin females than to the control side (solvent only) (extract, 0.59 ± 0.04; control, 0.40 ± 0.04, *n* = 134; P = 0.0370; Wilcoxon matched-pairs signed rank test). We then compared the GC profiles obtained from hexane extracts from males and females, but there was no evident difference between male and female traces. We fractionated the male and female extracts using silica gel columns and mixtures of hexane and diethyl ether, and then compared each female and male fraction. A close examination of the fractions obtained with 20% diethyl ether in hexane showed a minor peak at ca. 24.29 min, which was more intense in the female than in the male fraction (Fig. S2). The mean area of the peaks obtained with 5 extracts from females and 5 extracts from males and by injecting on a gas chromatograph (GC) 4 insect-equivalents showed a significant difference (Female extract, 582.7 ± 36.3 units of area (u.a.); Male extract, 347.9 ± 51.6 u.a.; Fig. S3) and confirmed that this compound was enriched in females as compared to males. Gas chromatography-mass spectrometry (GC-MS) analysis suggested, and comparison with authentic compound confirmed, that the female-enriched compound was tetracosyl acetate (=lignoceryl acetate). Lignoceryl acetate has been previously identified from 10 hymenopteran species, as well as a constituent of the pheromone gland of the leaf-rolling tortrix, *Sparganothis pilleriana* ^22^. In chemical ecology’s simplified nomenclature for pheromones, lignoceryl acetate is referred to as 24Ac so as to indicate that this semiochemical is an acetate made from a straight-chain, 24 carbon-long alcohol (lignocerol, 24OH).

### Field Evaluations of Lignoceryl Acetate-baited Traps

We tested in the field whether a commonly used trap to monitor ACP populations, ie, the yellow sticky traps^5,23–25^, baited with 24Ac at various doses would capture more ACP than control traps would capture. Although captures in traps baited with 24Ac at the doses tested (1, 10, and 100 µg per trap) in the first weeks of the experiments were not significantly different from the catches in control traps, we noticed a peculiar outcome for 24Ac-baited traps 5 weeks after traps were brought to the field (Fig. S4). We then repeated these experiments and obtained similar and consistent results (Fig. S5). Considering that the responses of these “old lures” were not dose-dependent (Figs S4 and S5), we repeated these experiments with the lowest dose only, ie, 1 µg per trap. Again, catches in 24Ac-baited traps were not different from those in control traps during the first weeks of the field tests, but significantly more males were captured in 24Ac-baited traps than in control traps at 35 and 42 days; thereafter the lures lost activity (Fig. S6). Behavioral measurement in our olfactometer using virgin males as responders and 24Ac as odor source, at a range of concentrations from 0.1 to 100 µg, did not show any significant preference for the treatments as compared to their respective controls (0.1 µg, *n* = 121, P = 0.2031; 1 µg, *n* = 197, P = 0.8867; 10 µg, *n* = 152, P = 0.9999; 100 µg, *n* = 121, P = 0.4671). We then surmised that, albeit chemically stable, 24Ac might undergo slow degradation under field conditions and that the active lure might be 24Ac degradation product(s). To test this assumption, we evaluated 24OH in the field and did not find significant differences between treatment and controls at doses from 0.1 to 100 µg (Dunnett’s multiple comparisons to control traps at each dose, each week, P = 0.2770–0.9999; *n* = 4 per dose per week). We then concluded that 24OH is not an ACP attractant. Next, we collected with solid phase microextraction (SPME) volatiles from lures matured for 5 weeks under field conditions. We detected small amounts of acetic acid in aged but not in new lures thus suggesting that 24Ac may have undergone degradation into 24OH and acetic acid. We also matured lures in a greenhouse and brought them to the field after 4 weeks. To decouple the trap color factor from chemical attraction, we used in these experiments transparent traps baited with matured 24Ac lures or solvent only. Traps loaded with 24Ac lures aged for 35 days collected significantly more males than control traps collected in the first week (Fig. S7) but lost all activity after 3 weeks (Fig. S7). These findings suggest that acetic acid might be an attractant.

### Male and Female Release of and Behavioral Responses to Acetic Acid

**Figure 4:**
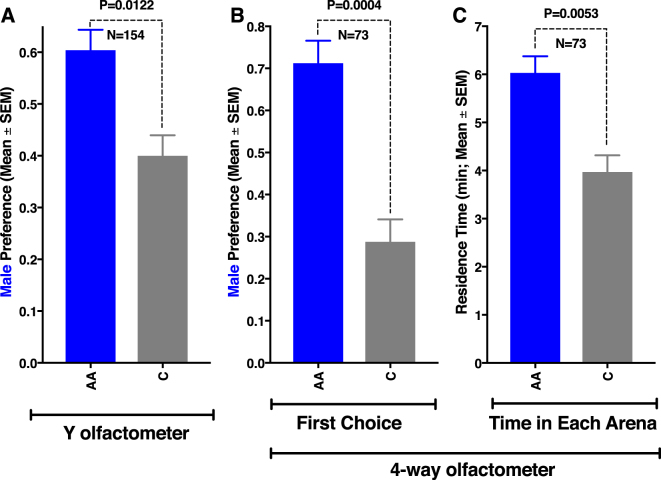
Behavioral responses of 7-day-old virgin males to acetic acid (AA). Responses in a (**A**) Y-olfactometer, and a (**B** and **C**) 4-way olfactometer. In both assays (**A** and **B**), males were significantly attracted to the AA side of the arena. They also spent significantly more time (**C**) on the AA than in control area.

Acetic acid has been previously identified in extracts from ACP females^14^, but its function was not ascribed or the synthetic compound tested. SPME-based volatile collections from separated groups of 50 ACP males and females, which were removed from the host plant at least 12 h prior to the headspace collections (extracts), and subsequent GC-MS analyses showed that female extracts contained acetic acid (Fig. S8), although trace amounts were also detected in male extracts. Recently, acetic and formic acids were identified as degradation products from plant volatile cartridges prepared for electrophysiological studies^26^ and a tertiary blend, including these acids plus p-cymene, was demonstrated to be a phagostimulant, but not an attractant to ACP^27^. On the other hand, acetic and propionic acids were reported to inhibit responses from olfactory receptor neurons in ACP antennae^28^. We recorded EAG responses from ACP male and female antennae using a series of acids from 1–6 carbons. Male antennae generated robust EAG responses to acetic acid (2.70 ± 0.36 mV, *n* = 26) and propionic acid (2.22 ± 0.29 mV), moderate responses to formic acid (1.60 ± 0.63 mV), weak responses to butyric acid (0.52 ± 0.36 mV) and valeric acid (0.24 ± 0.03 mV), and no response to hexanoic acid when tested at a high dose (source dose, 10^−1^). At a lower dose (10^−2^), only acetic (1.73 ± 0.37 mV, *n* = 19), propionic (1.13 ± 0.47 mV), and formic (0.42 ± 0.37 mV) acids responded. Likewise, female antennae had stronger responses to acetic acid (source dose, 10^−1^: 3.22 ± 0.94 mV, *n* = 15) than to propionic acid (2.90 ± 1.09 mV) and formic acid (1.86 ± 0.48 mV). In summary, both male and female antennae generate robust responses to acetic acid, low responses to formic acid, and moderate responses to propionic acid, particularly at higher doses. At very low doses (10^−3^), only male antennae responded to acetic acid (0.44 ± 0.21 mV). Of note, there was no need to subtract background responses given that blank EAG was almost indistinguishable from the baseline. In light of these EAG results, we re-analyzed the SPME collections from male and female to determine whether other EAG-active acids were also released by ACP males and/or females. Again, acetic acid was easily detected, particularly in female airborne volatile collections at the time corresponding to the peak of mating activity, but propionic and formic acids were not detected. Next, we used both a Y-tube olfactometer and an air-flow olfactometer,^29^ also known as “4-way olfactometer,” to measure ACP olfactory responses to acetic acid. Males were significantly more attracted to the acetic acid side of the Y-olfactometer than to the control side (Fig. 4A). Male preference for the acetic acid-laden fields in the 4-way olfactometer as compared to the control sides was highly significant (Fig. 4B). Additionally, males spent significantly more time in the acetic acid than in the control fields (Fig. 4C). Taken together, these behavioral measurements suggest that acetic acid is a male attractant.

### Field Evaluations of Acetic Acid-baited Traps

**Figure 5:**
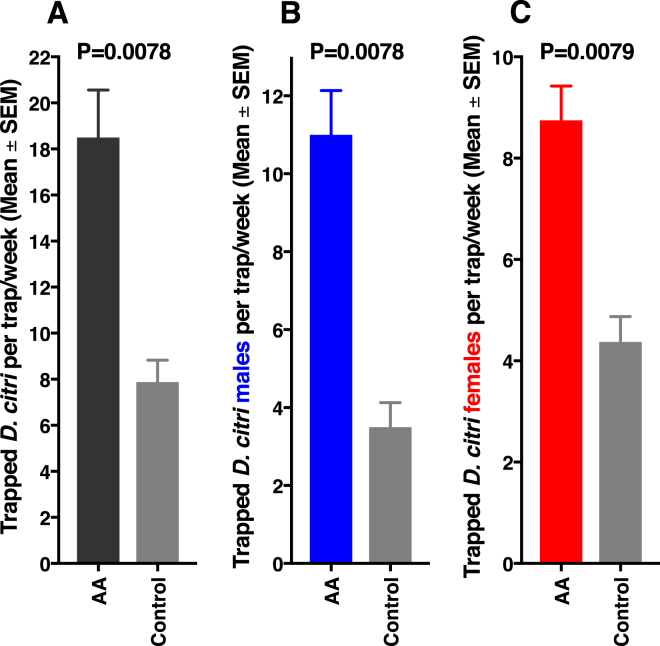
Results of field tests with yellow sticky traps baited either with acetic acid (AA) or hexane (control). (**A**) Mean number of adults captured per trap per week. (**B**) and (**C**) mean numbers of males and female caught per trap per week, respectively.

**Figure 6:**
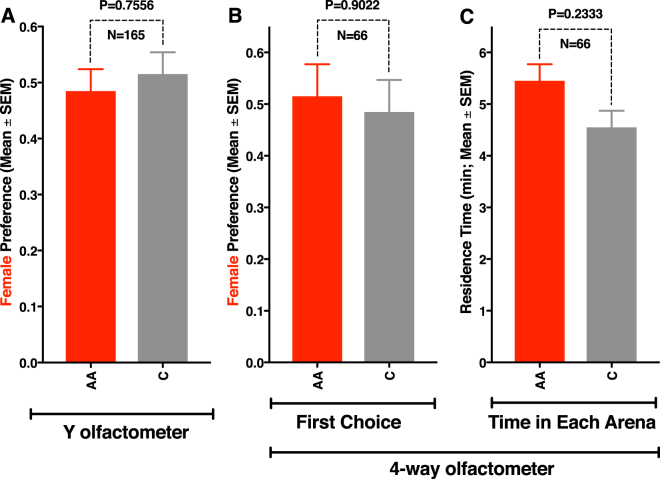
Behavioral responses of 7-day-old virgin females to acetic acid (AA). Responses in a (**A**) Y-olfactometer, and a (**B** and **C**) 4-way olfactometer. In both assays (**A** and **B**), males were significantly attracted to the AA side of the arena. They also spent significantly more time (**C**) on the AA than in the control area.

Traps baited with acetic acid (1 µg per trap) captured significantly more ACP adults than control traps captured (Fig. 5A). Surprisingly, captures were significantly higher not only for ACP males (Fig. 5B), but also for females (Fig. 5C). Additional preliminary experiments showed even better trap performance at higher doses. Control traps captured 1.48 ± 0.22 males/trap per day, whereas traps baited with 1, 10, and 100 µg of acetic acid caught significantly (*n* = 48 per trap; Dunnett’s multiple comparisons) more insects, ie, 2.48 ± 0.25 (P = 0.0022), 3.08 ± 0.33 (P = 0.0001), and 4.23 ± 0.35 males per trap per day (P = 0.0001), respectively. Likewise, traps baited with acetic acid captured more females than control traps captured (control 1.08 ± 0.14 females/trap per day; 1, 10, and 100 µg: 1.5 ± 0.21 (P = 0.4880), 1.65 ± 0.20 (P = 0.1435), and 2.33 ± 0.24 females/trap per day (P = 0.0001), respectively. These findings suggest that acetic acid is an aggregation-sex pheromone^30^, ie, a semiochemical produced by one of the sexes that attracts both sexes for mate procurement. We tested this assumption by measuring female behavior in our olfactometers, which provides a better assessment of attraction. In marked contrast to what has been observed with males (Fig. 4), response of females to the arm of the Y olfactometer baited with acetic acid did not differ significantly from the response to the control side of the arena (Fig. 6). Likewise, females did not distinguish between the treatment and control sides of the 4-way olfactometer (Fig. 6B). Furthermore, the time spent on the treatment and control sides were not significantly different (Fig. 6C). We, therefore, concluded that acetic acid is not a female attractant. We surmise that the significantly higher captures in acetic acid-baited traps (eg, Fig. 5) might be due to other factors, such as chemical or physical signals elicited by captured males. Considering that males did not attract females in our indoor assays (Fig. 3B), it is more likely that physical stimuli from captured males may be involved in the observed higher catches of females in acetic acid-baited traps. As a matter of fact, vibrational communication in ACP is well-documented in the literature^15,31^ and has been explored as a tool for ACP trapping^32^ and mating disruption^33^. Regardless of the mode of action, the fortuitous discovery that a female-derived sex pheromone ultimately leads to capture of both males and females provides a better tool for monitoring ACP populations.

## Materials and Methods

### Insect Preparations

A laboratory ‘*Ca*. Liberibacter spp.’ free colony was initiated with insects collected from *M*. *paniculata* in São Paulo State, Brazil in 2009 and reared in a greenhouse under 25 ± 5 °C. For this purpose, 400 mated adults were placed in cages (60 × 60 × 60 cm) containing *M*. *paniculata* seedlings and kept for 7 days for oviposition. Then, adults were removed and the seedlings with eggs were kept in cages. Once nymphs reached the fifth instar, cages were transferred to a climate-controlled room at 25 ± 2 °C, 65 ± 10% RH, and L14:D10 h photoperiod until adult emergence. These conditions were selected to mimic field conditions during ACP flight activity in São Paulo, Brazil. Luminosity was set at 3000 lux. Newly emerged adults were collected and separated by sex for bioassays.

### Mating behavior analysis

These experiments were performed in a climate-controlled room at 25 ± 2 °C, 65 ± 10% RH, L14:D10 h photoperiod, and 3000 lux luminosity. For this purpose, *M*. *paniculata* seedlings were pruned 5-cm height 15 days before the beginning of the experiment to allow emission of flushes. Seedlings containing flushes of ~2 cm in length were allocated to plastic cages 10 cm in height × 7.5 cm in diameter and used as an experimental unit. After that, one virgin *D*. *citri* male and one virgin female (couple) with 1, 2, 3, 4, 5, 6, and 7 days after emergence (treatments) were released inside the cage. For each treatment (couple age), 70 replicates were performed. The mating activity of each couple were assessed every 30 min (24 h a day) for 7 days.

### Indoor behavioral assays

All behavioral assays were conducted in a climate-controlled room (see above). A glass tube in a Y-shape (central tube 15 cm in length and 3 cm in diameter split into two tubes in a 120^o^ angle with the same dimensions used in the central tube) was placed vertically with the decision arms pointing towards the ceiling (light sources). All connections were made with 0.635-diameter polytetrafluoroethylene (PTFE) tubes (Sigma-Aldrich, Bellefonte, PA, USA). Clean air was provided by an oil-free air compressor (Schulz MSV6, Schulz, Joinville, SC, Brazil) and humidified into milliQ water before the line was split into two separate lines, each one with an air flowmeter with a range from 0.1 to 1.0 L/min (Brooks Instrument, Hatfield, PA, USA) to allow balanced flow of 0.4 mL/min. Treatment (eg, acetic acid 0.01 µL/µL hexane 100 µL) and control (eg, 100 µL hexane) were placed on cotton inside glass chambers (20 cm in length × 6 cm in diameter) with one end connected to one of the airflow lines and the other end connected to one of the decision arms of the olfactometer. Each replicate was performed with one insect (7-day-old virgin male or female) being place inside the downwind arm of the arena, which was covered with a glass lid. Insects that crossed the central tube line located 5 cm from the bifurcation within the observation time of 5 min were recorded as responders. After that, 5 min were allowed for the responder insect to cross the lateral tube lines, located 5 cm from the connection with the central tube, allowing observation of the choice of odor. An olfactometer test consisted of the release of a single *D*. *citri* and the observation (from 15:00 to 18:00).

An air-flow olfactometer, also known as “4-arm olfactometer” or “4-way olfactometer” was constructed^29^ (30.0 × 30.0 × 2.5 cm in length, width and height, respectively) with acrylic. A constant, charcoal-filtered humidified airflow 0.1 L/min was connected to each odor source, and all airflows converged through individual PTFE tubes to the center of the acrylic arena. Yellow fields were made by adding a yellow laser jet printed-paper below the bottom of the arena with the following color spaces: lightness (84.8 ± 0.04), chroma (98.7 ± 0.40), and *hue* angle (95.7 ± 0.02) using a colorimeter Minolta CR400 (Konica Minolta Co., Osaka, Japan)^34^. Two of the four possible arms received volatiles from acetic acid 0.01 µL/µL hexane (100 µL), whereas the other two remaining received hexane. The psyllids (virgin 7-day-old males or females) were released on the center of the arena. An olfactometer test consisted of the release of a single *D*. *citri* and the observation (from 15:00 to 18:00). As a criterion for data collection from each insect, five minutes were allowed for response (first choice). In case of response, 10 minutes were allowed to observe the time spent in each of the four odor fields.

### Field Experiments

#### Experiment 1: Evaluations of lignoceryl acetate, lygnocerol, and acetic acid

These experiments were performed in an organic grove of “Valência” sweet orange [*Citrus sinensis* (L.) Osbeck] grafted on ‘Rangpur’ lime (*Citrus limonia* Osbeck) with natural infestation of *D*. *citri*. For this purpose, yellow stick cards (30 cm in length × 10 cm in width) with a 2 cm in diameter central hole were used to assess the attractivity of the test compounds to *D*. *citri* adults. All compounds were assessed at 1, 10, and 100 µg. Hexane was used as a control. Tested compounds were incorporated into slow-release devices made of ES fiber (Ethylene-Propylene Side by Side, Chiso Co. Ltd, Japan). The experiments were designed in Latin square using 16 traps spaced 25 m from each other (4 traps per treatment). They were repeated three times over time and the number of captured ACP adults was recorded each 7 days for 49 days.

#### Experiment 2: Evaluation of aged lignoceryl acetate lures

Lures were prepared and kept in greenhouses for 28 days prior to being fixed in yellow stick cards in the field. Thus, aged lures were tested in the field starting on day 28 until 70 days after preparation of the lures. The area, experimental design, number of traps, and data collection were the same as described above.

#### Experiment 3: Evaluation of lignoceryl acetate lures using transparent traps

To avoid interference from the yellow visual cues from the stick cards, these experiments were performed with transparent acrylic traps (30 cm in length × 10 cm in width) and covered with an adhesive glue film (Biostop Cola, Biocontrole, São Paulo, Brazil). The experimental design, number of traps, and data collection were the same as described above.

### Statistical analyses

All statistical analyses were done with Prism 7 (GrapPad, La Jolla, CA). Each pair of a test and a control in indoor behavioral measurements using an Y-olfactometer or 4-way olfactometer, as well in field tests comparing only a single treatment and a control, was compared by using two-tailed, Wilcoxon matched-pairs signed rank test. Field tests with multiple comparison (different doses) were analyzed by ANOVA. Capture data were first transformed into log (x + 1) and them means were analyzed by Dunnett’s test as well as Tukey’s test (P < 0.05) by comparing the mean of each column with the mean of the control or with the mean of every other group, respectively.

### Electrophysiology

Antennas were mounted, according to a published protocol^26^, under a stereoscopic microscope (SZT, BEL Engineering, MB, Italy). Antennae were connected with glass microcapillaries pulled with a PC-10 puller (Narishige, Kanto, Japan). The glass capillaries housed 0.39 mm gold wires (Sigmund Cohn Corp, Mt. Vernon, New York) and were filled with a saline solution (3.7 g NaCl, 0.175 g KCl, 0.17 g CaCl_2_ in 500 mL of distilled water). Electrodes were connected to a Universal Probe, gain 10X (Syntech, Buchenbach, Germany) and connected to a 2-Chanel IDAC Acquisition Controller (Syntech). The signal was processed with Syntech software (GcEad version 4.6). Two Pasteur pipettes were used one for a flow balance and the other as a stimulus. The stimulus was loaded on a filter paper strip (40 × 4-mm), which was inserted in the stimulus pipette after evaporation for 1 min. Each cartridge was used only once. The antennal preparation was set at the end of an effluent flow tube, which had two lateral holes (13 cm from the antennal preparations) to accommodate the tips of the Pasteur pipettes (stimulus and flow balance). Humidified continuous flow (0.1 L/min) was generated by a CS-55 Stimulus Controller (Syntech). Flow for stimulus and balance were set at 1 L/min. Stimulus duration was set a 1 s. There was a minimal interval of 1 min between stimuli.

### Chemical Analyses

In Davis, GC-MS was performed on a 5973 Network Mass Selective Detector linked to a 6890 GC Series Plus + (Agilent Technologies, Palo Alto, CA). The GC was equipped with an HP-5MS capillary column (30 m × 0.25 mm; 0.25 µm film; Agilent Technologies), which was operated with the following programs: 70 °C for 1 min, subsequently increased at a rate of 10 °C/min to 270 °C, and held at this final temperature for 10 min, ie, 70(1)−10-270(10), and 50(4)-5-200(1)-10-270(10) for analysis of extracts and fractions. The injector was operated at 250 °C in pulsed splitless mode. MS transfer line was set at 280 °C, and the MS quad and MS sources were set at 150 °C and 230 °C, respectively. In Piracicaba, GC-MS was performed on a Varian CP-3800 gas chromatograph fitted with an HP5-MS capillary column (30 m × 0.25 mm i.d. × 0.25 µm film; J&W Scientific, Folsom, CA), coupled to a 4000 Ion Trap Mass Spectrometer (Varian, CA, USA) and on a Shimadzu GCMS-QP2010 Ultra linked to a Shimadzu GC-2010 Plus (Shimadzu Corp., Kyoto, Japan) fitted with an RTx-1MS capillary column (30 m × 0.25 mm i.d. × 0.25 µm film; Restek Corporation, Bellefonte, PA, USA). Injections were operated in splitless mode with an injector temperature of 250 °C. The GC ovens were programmed from 40 °C for 1 min, increased to 300 °C at 10 °C/min (held for 50 min). MS transfer line, manifold, and trap were set at 300 °C, 50 °C, and 217 °C, respectively. Quantitative analyses were done on a Shimadzu GC- 2010 gas chromatograph equipped with a flame ionization detector (Shimadzu Corp., Kyoto, Japan) and fitted with an HP5-MS capillary column (30 m × 0.25 mm i.d. × 0.25μm film; Agilent Scientific, Santa Clara, CA, USA). SPME analyses were done with a 50/30 divinylbenzene (DVB)/carboxen (CAR)/polydimethylsiloxane (PDMS) (Supelco, catalogue number 57328-U). Volatile compounds were collected from the headspace of the samples placed in a closed glass container with a rubber septum on the top. Both laboratories used the same oven temperature program, ie, starting at 35 °C for 5 min, raising to 70 °C at a rate of 2.5 °C/min, then increasing to 150 °C at 5 °C/min, and subsequently raising to the final temperature of 250 °C at 20 °C/min.

### Airborne Volatile Collections, Crude Extracts, and Fractionations

Airborne volatile collections were done in all-glass chambers by passing clean air (as described above), trapping volatile compounds on Tenax TA (30–60 mesh, Sigma-Aldrich), and extracting them with hexane or pentane. Whole body extractions were obtained by washing with hexane for 3 min batches of 1,000–5,000 virgin males and female. The solvent was then transferred to a clean vial, a small aliquot of hexane was added, the solvent transferred, and the last step was repeated one more time. The combined volume of the three washes was adjusted to generate male and female extracts with equal number of insect-equivalents per volume (typically 1 or 4 insect-equivalents per µl). Crude extracts (3,000 female- or 3,000 male-equivalents) were subjected to flash column chromatography on silica gel (60–200 mesh; Fisher Scientific, Pittsburgh, PA, US) by successively eluting with hexane-ether mixtures in the following order: 100:0 (hexane fraction), 95:5 (5% fraction), 80:20, 50:50, 0:100 (ether fraction). Crude extracts and fractions were analyzed by GC and GC-MS for comparison of male and female profiles.

### Chemicals

Formic, acetic, propionic, butyric, valeric, and hexanoic acids, hexane, lignoceryl alcohol, acetic anhydride, pyridine, and anhydrous sodium sulfate were purchased from Sigma-Aldrich (Milwaukee, WI). Anhydrous dichloromethane (DCM) was acquired from ACROS (New Jersey, US). An aliquot of 11 mg of lignoceryl acetate was prepared in two batches, one starting with 10.2 mg and the other with 7 mg of lignocerol dissolved in anhydrous DCM. A pyridine/DCM solution was added stepwise; the reactions were left at room temperature for 5 h. Then, 0.5 M HCl was added, the organic phase was extracted and washed with brine, and anhydrous sodium sulfate was added. After reducing the volume by evaporation, the crude material was subjected to flash column chromatography (see above). An aliquot (1 mg) was used to make a hexane solution for GC-MS analysis and comparison of retention times. The rest of the sample was used for indoor behavioral assays and field tests in Brazil.

## Electronic supplementary material

Supplementary Information
