# Supplementary material for: Putative sex pheromone of the Asian citrus psyllid, Diaphorina citri, breaks down into an attractant
Source: Sci Rep. 2018 Jan 11;8:455. doi: 10.1038/s41598-017-18986-4 (PMC5764970; doi:10.1038/s41598-017-18986-4)
Supplement: Supplementary file 1 — Supplementary Information [file 41598_2017_18986_MOESM1_ESM.pdf]

Supplementary Information for

Putative sex pheromone of the Asian citrus psyllid, *Diaphorina citri*, breaks down into an attractant

Odimar Z. Zanardi, Haroldo X. L. Volpe, Arodi P. Favaris, Weliton D. Silva, Rejane A. G. Luvizotto, Rodrigo F. Magnani, Victoria Esperança, Jennifer Y. Delfino, Renato de Freitas, Marcelo P. Miranda, José Roberto P. Parra, José Mauricio S. Bento, and Walter S. Leal

Correspondence to: wsleal@ucdavis.edu

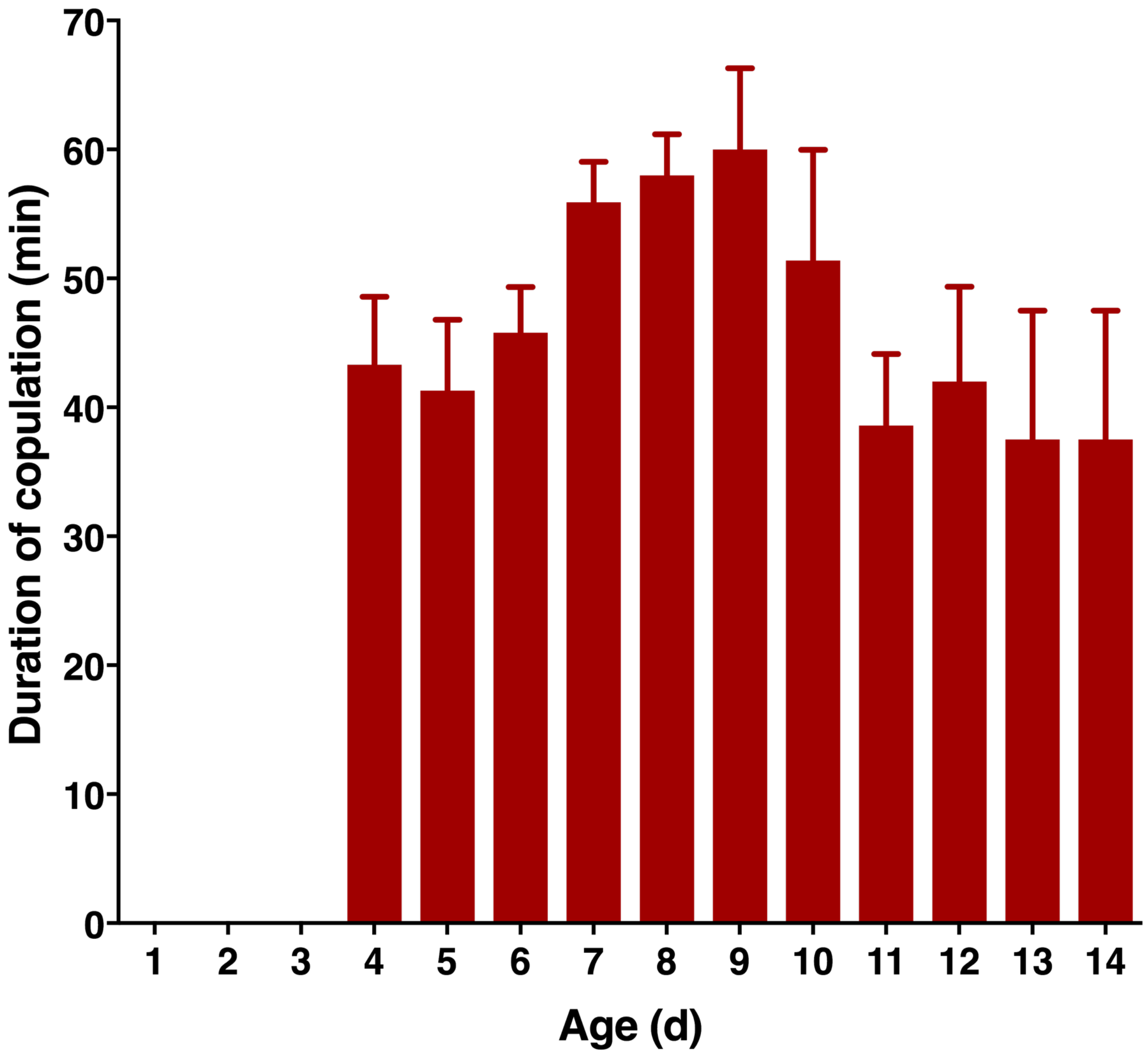

Figure S1. Duration of copulation (Mean±SEM, min) according to insect age.

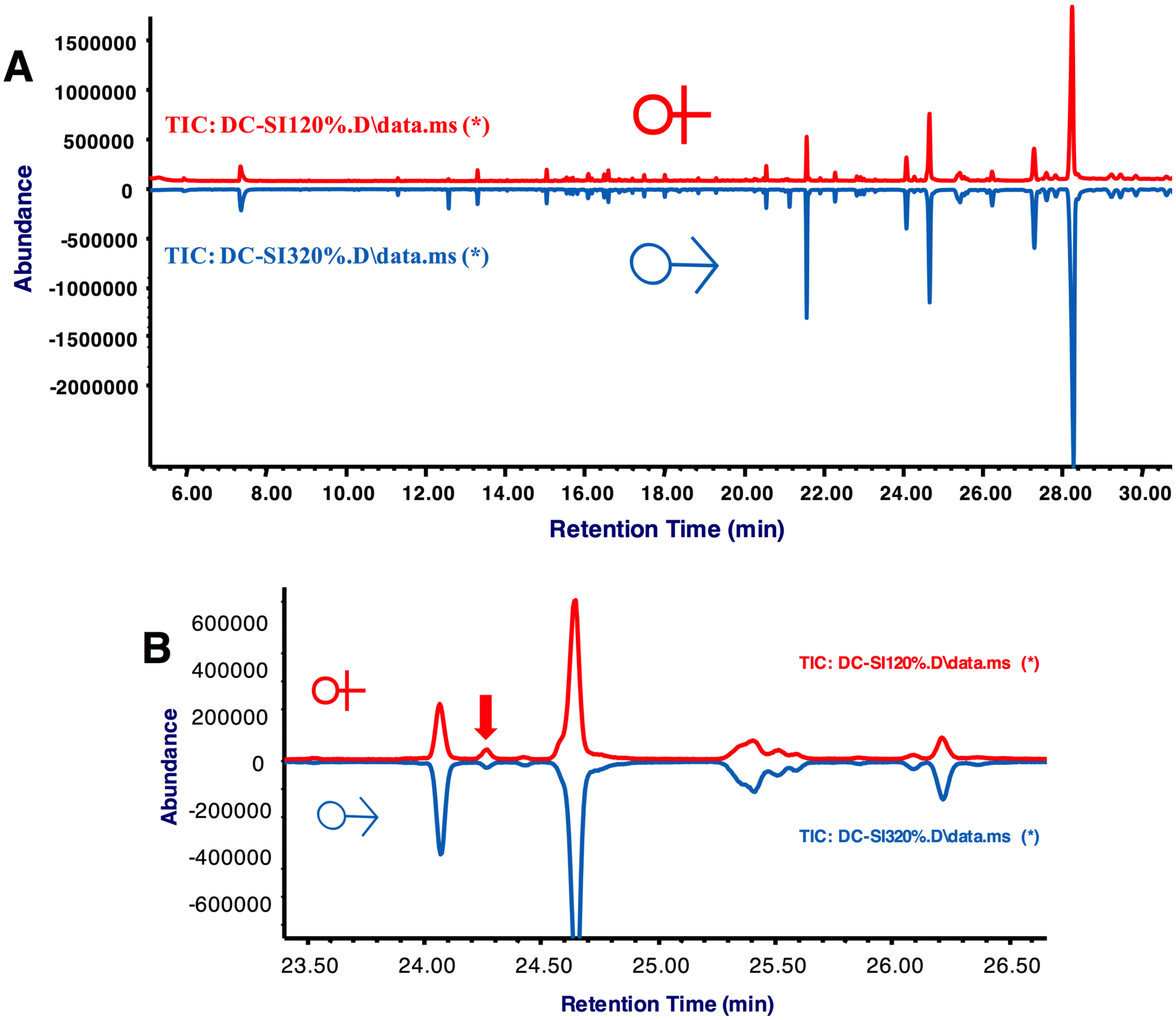

Figure S2. Chromatograms from a fraction obtained after fractionating females (red) and male (blue) whole-body extracts. (A) Expanded view and (B) highlight of an area of the chromatograms showing a difference (indicated with an arrow) between males and females. The female-enriched peak was identified as lignoceryl acetate.

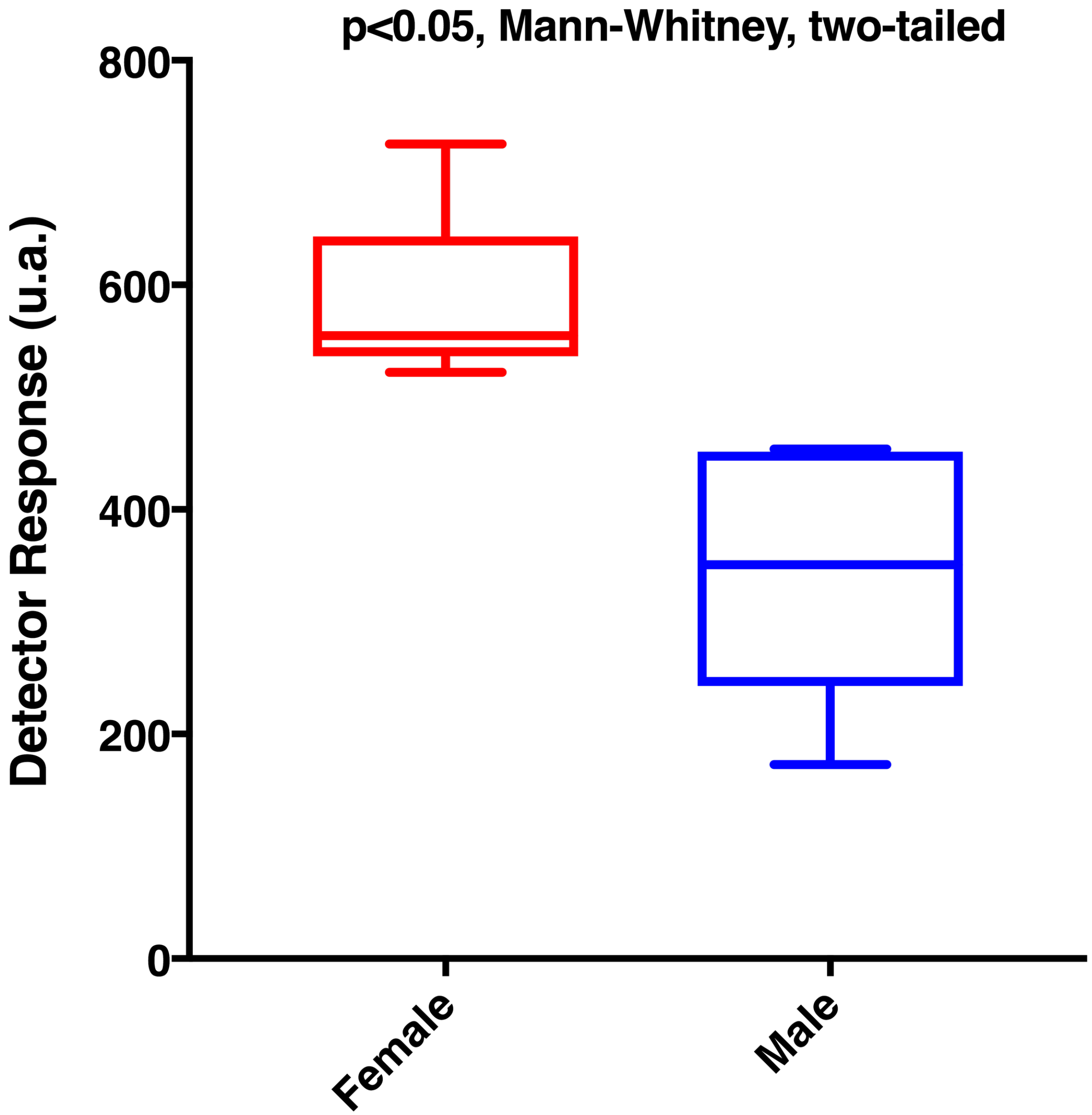

Figure S3. Results of quantification of the areas of the peak highlighted in the previous figure. Crude extracts (4 adult-equivalent) from males and females were injected (n = 5) for quantification. Female extracts (red) contained significantly more lignoceryl acetate than male extracts contained (blue).

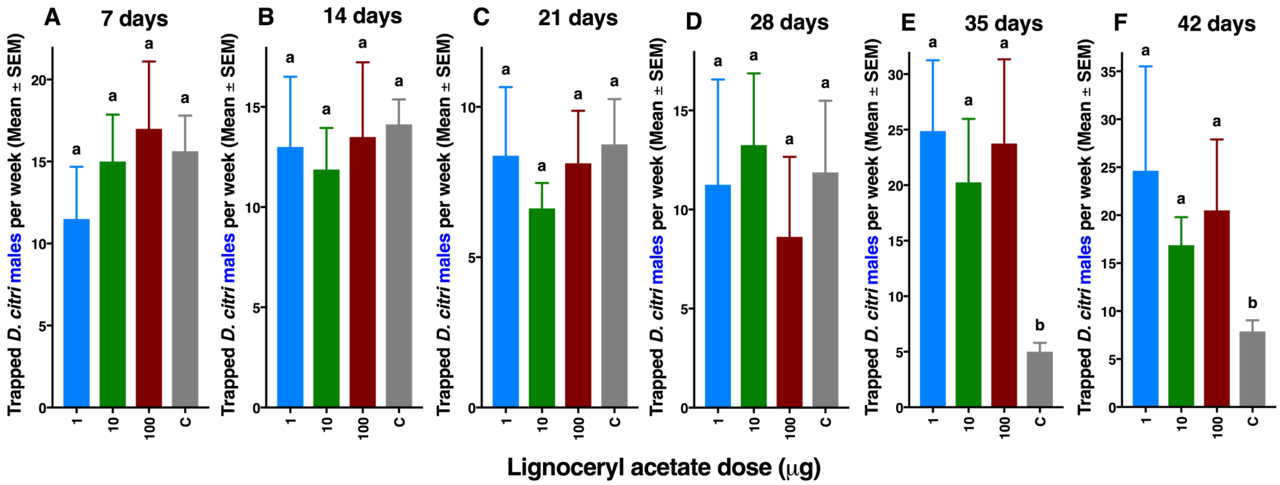

Figure S4. Field test data to evaluate lignoceryl acetate at various doses. Results obtained after (A) 7, (B) 14, (C) 21, (D) 28, (E) 35, and (F) 42 days. Traps were replaced weekly, but the lures were maintained for the entire duration of the experiment. Typically, experiments are terminated after a short duration showing negative results, but the extended time shows a peculiar performance after 4 weeks. Although captures in 24Ac-loaded traps were not significantly different from those in control traps until 28 days, a significant difference was observed at 35 and 42 days. Column with the same letters are not significantly different.

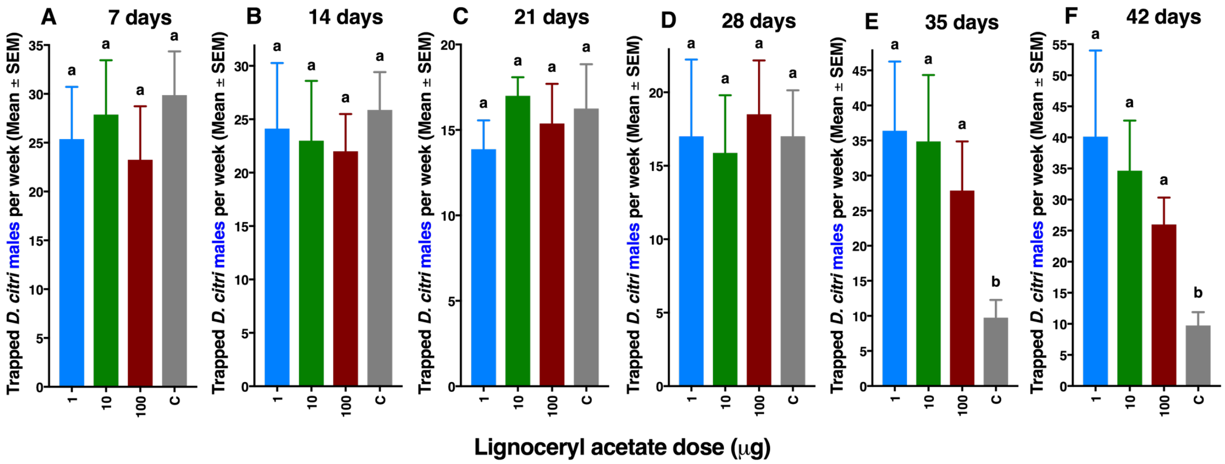

Figure S5. Results of field test to evaluate lignoceryl acetate at various doses. Results obtained after (A) 7, (B) 14, (C) 21, (D) 28, (E) 35, and (F) 42 days. Captures in 24Ac-baited traps were significantly higher than captures in control traps 35 and 42 days after the onset of the experiments, but not earlier.

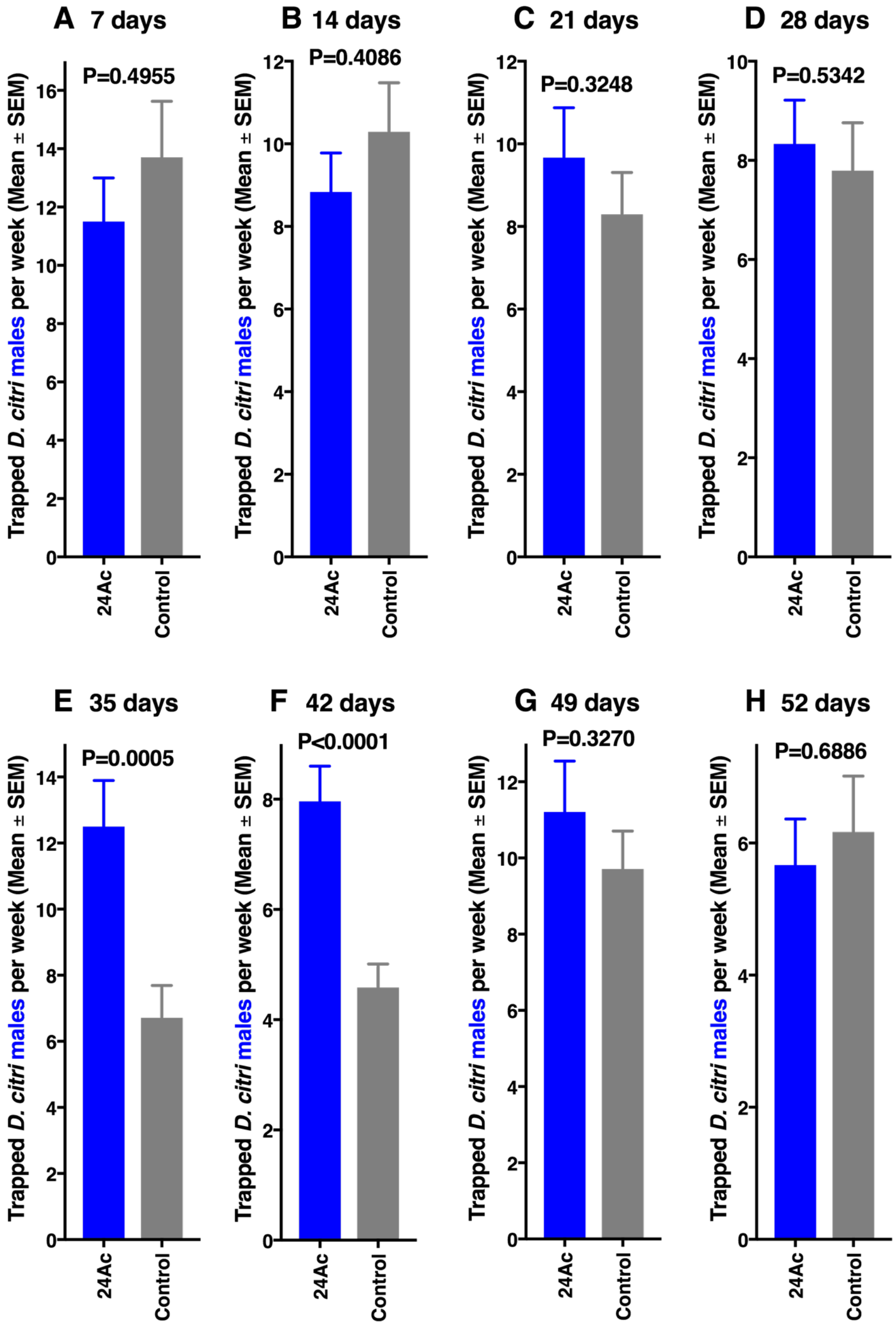

Figure S6. Results of field tests to evaluate lignoceryl acetate at a low dose. Results obtained after (A) 7, (B) 14, (C) 21, (D) 28, (E) 35, and (F) 42, (G) 49, and (H) 52 days. Captures in 24Ac-baited traps were significantly higher than captures in control traps 35 and 42 days after the onset of the experiments, but not earlier or after that time window.

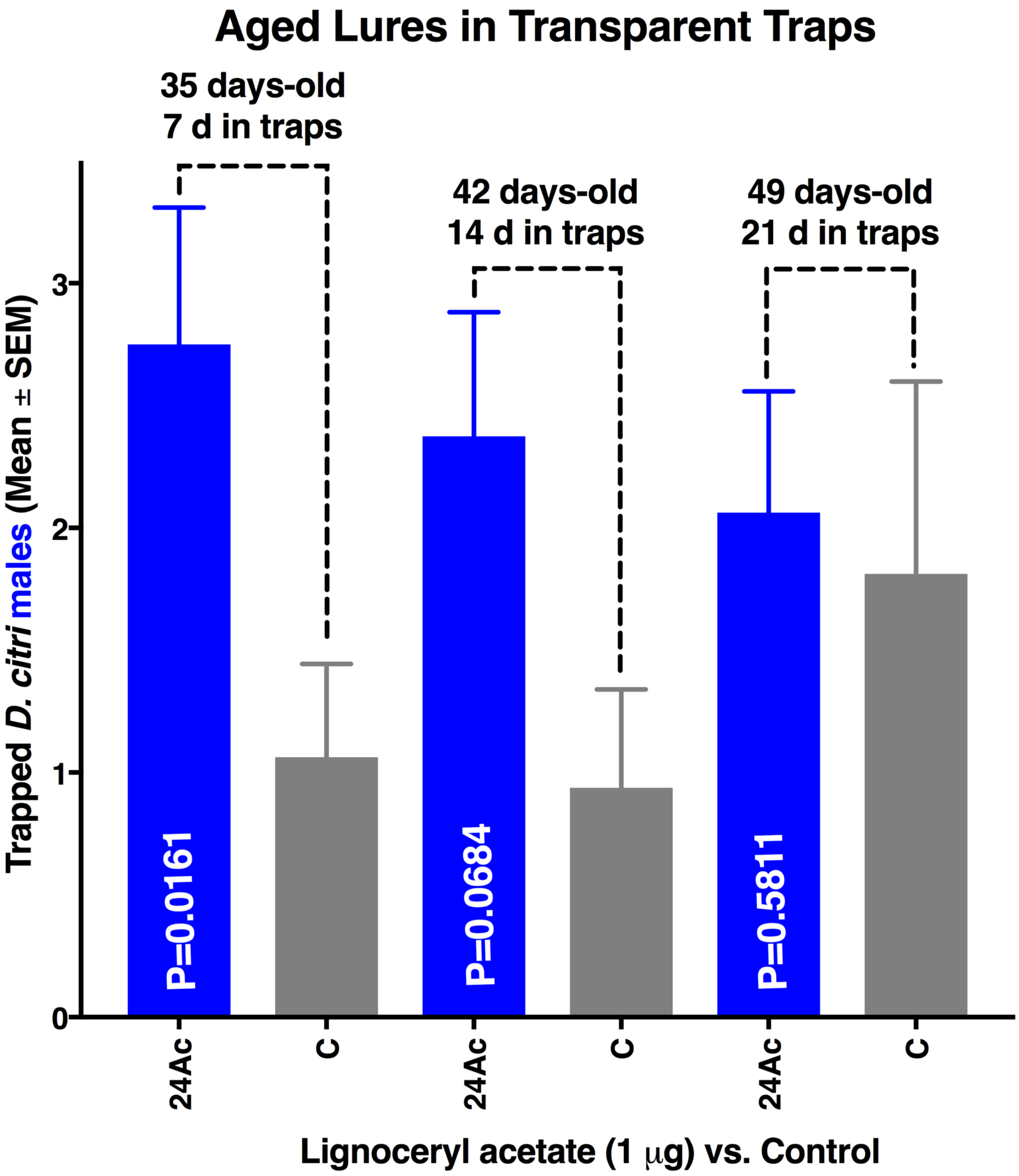

Figure S7. Results of field tests with transparent traps and aged lures. Captures in traps with aged lures were significantly higher than catches in control traps during the first week in the field. In the second week, captures decreased, and in the third week after being matured and deployed in the field, the lures lost activity.

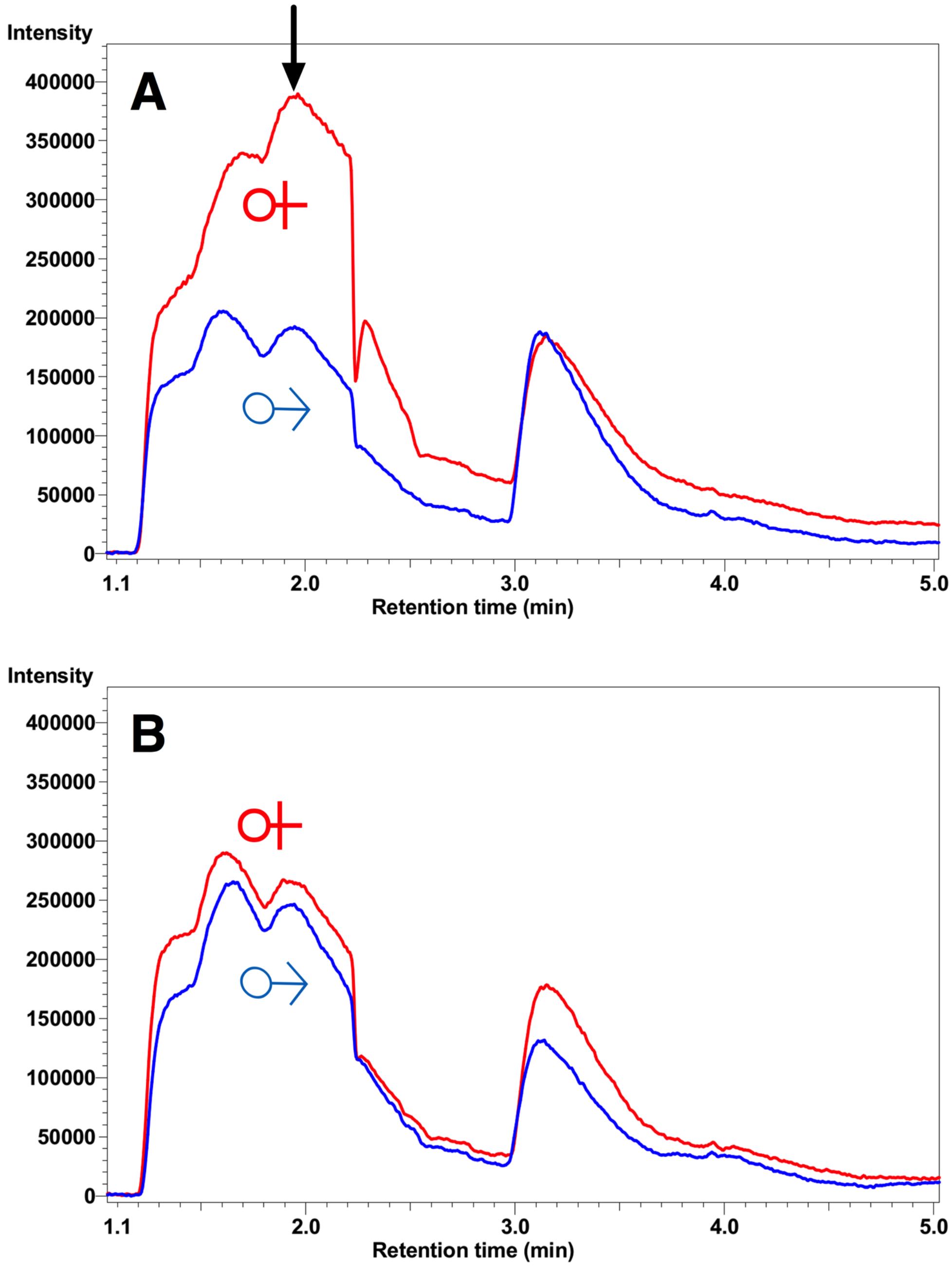

Figure S8. Chromatograms from SPME analyses from ACP virgins, 7-day-old males (blue) and females (red). (A) Airborne volatile capture during the time corresponding to the window of mating activity. (B) Similar collections obtained from males and females during the scotophase when no mating activity takes place. An arrow highlights the peak of acetic acid.
